# Wnt/β-catenin signaling activation promotes lipogenesis in the steatotic liver via physical mTOR interaction

**DOI:** 10.3389/fendo.2023.1289004

**Published:** 2023-12-13

**Authors:** Kewei Wang, Rong Zhang, Nadja Lehwald, Guo-Zhong Tao, Bowen Liu, Bo Liu, Yangseok Koh, Karl G. Sylvester

**Affiliations:** ^1^ Department of Surgery, Divison of Pediatric Surgery, Stanford University School of Medicine, Stanford, CA, United States; ^2^ Department of Gastrointestinal Surgery, First Hospital of China Medical University, Shenyang, Liaoning, China; ^3^ Department of General, Visceral and Pediatric Surgery, School of Medicine, Heinrich Heine University, Duesseldorf, Germany; ^4^ Department of Surgery, Chonnam National University Medical School, Gwangju, Republic of Korea; ^5^ Stanford Metabolic Health Center, Stanford University School of Medicine, Stanford, CA, United States

**Keywords:** Wnt signaling, hepatic steatosis, fatty acid synthesis, high fat diet, beta-catenin (B-catenin)

## Abstract

**Background and aims:**

Wnt/β-catenin signaling plays an important role in regulating hepatic metabolism. This study is to explore the molecular mechanisms underlying the potential crosstalk between Wnt/β-catenin and mTOR signaling in hepatic steatosis.

**Methods:**

Transgenic mice (overexpress Wnt1 in hepatocytes, Wnt+) mice and wild-type littermates were given high fat diet (HFD) for 12 weeks to induce hepatic steatosis. Mouse hepatocytes cells (AML12) and those transfected to cause constitutive β-catenin stabilization (S33Y) were treated with oleic acid for lipid accumulation.

**Results:**

Wnt+ mice developed more hepatic steatosis in response to HFD. Immunoblot shows a significant increase in the expression of fatty acid synthesis-related genes (SREBP-1 and its downstream targets ACC, AceCS1, and FASN) and a decrease in fatty acid oxidation gene (MCAD) in Wnt+ mice livers under HFD. Wnt+ mice also revealed increased Akt signaling and its downstream target gene mTOR in response to HFD. *In vitro*, increased lipid accumulation was detected in S33Y cells in response to oleic acid compared to AML12 cells reinforcing the *in vivo* findings. mTOR inhibition by rapamycin led to a down-regulation of fatty acid synthesis in S33Y cells. In addition, β-catenin has a physical interaction with mTOR as verified by co-immunoprecipitation in hepatocytes.

**Conclusions:**

Taken together, our results demonstrate that β-catenin stabilization through Wnt signaling serves a central role in lipid metabolism in the steatotic liver through up-regulation of fatty acid synthesis via Akt/mTOR signaling. These findings suggest hepatic Wnt signaling may represent a therapeutic strategy in hepatic steatosis.

## 1 Introduction

Fatty acid synthesis and oxidation in the liver are critical components of lipid homeostasis. Dysregulation of the balance between fatty acid synthesis and oxidation can lead to the accumulation of lipids within hepatocytes. Non-alcoholic fatty liver disease (NAFLD) is a series of progressive diseases caused by the accumulation of fat in the liver, characterized by increased hepatic triglyceride content in the absence of excessive alcohol consumption (1). In recent years, the prevalence of NAFLD is increasing globally, affecting nearly a quarter of the world’s population, and has become the leading cause of chronic liver disease worldwide (2, 3).

Wnt signaling plays multiple functions in liver development, physiology, pathology and especially liver zonation, the abnormities of which has been indicated in the pathogenesis of diet-induced fatty liver and obesity, potentially via regulation of endoplasmic reticulum stress and enzymes involving lipid metabolism (4–6). The canonical Wnt signaling cascade polymerizes on the transcriptional regulator β-catenin, with the end result being nuclear translocation of β-catenin (7). Research in the MacDougald lab was the first to report that Wnt signaling functions as an adipogenesis switch *in vitro*. Specifically, they suggest that Wnt-10b is an endogenous regulator of adipogenesis, and overexpression of Wnt1 in 3T3-L1 preadipocytes can inhibit adipogenesis (8). Wnt1-overexpressing mice was constructed by our team, and we found that Wnt1 overexpression confers significant hepatic protection against ischemia-reperfusion injury (9). Previous studies have shown that persistent activation of the canonical Wnt signaling pathway in preadipocytes *in vivo* can also inhibit adipocyte differentiation, resulting in complete fibrosis of subcutaneous adipose tissue (10). However, recent studies have found that persistent activation of the canonical Wnt pathway leads to the accumulation of fat (4, 6, 11). These researches suggest that the activation of canonical Wnt signaling has regional differences in different adipose tissues, but the exact mechanism remains unclear.

Mammalian target of rapamycin (mTOR) is a serine/threonine protein kinase which downstream of phosphatidylinositol 3-kinase (PI3K)/protein kinase B (Akt) that acts as a central regulator of metabolism and can integrate a variety of nutritional and hormonal signals to control anabolic processes. The two major catalytic subunits of mTOR kinase, mTOR complex 1 (mTORC1) and mTOR complex 2 (mTORC2), provide NADPH necessary for lipid synthesis through regulation of pentose phosphate pathway. Activation of mTOR was associated with increased lipid synthesis and lipid droplets, whereas inhibition of mTOR resulted in higher rates of oxidation (12). Evidence has shown that KIF2C is a direct target of the Wnt/β-catenin pathway, and acts as a key factor in mediating the crosstalk between Wnt/β-catenin and mTORC1 signaling in hepatocellular carcinoma (13). These observations indicate that mTOR signaling may interact with the Wnt/β-catenin pathway in some way. However, no prior studies have explored the molecular mechanisms underlying the potential crosstalk between Wnt/β-catenin and mTOR signaling given a chronic metabolic exposure and injury like high fat diet (HFD). Since both Wnt/β-catenin and mTOR signaling have established roles in regulating hepatic metabolism, the present study was designed to elucidate the relevance and molecular mechanisms governing possible β-catenin/mTOR interactions in the clinical setting of hepatic steatosis in a Wnt/β-catenin activated murine *in vivo* and *in vitro* model under homeostatic conditions and in response to HFD.

## 2 Materials and methods

### 2.1 Transgenic mice model

Wnt1+ double transgenic mice were generated to overexpress Wnt1 in hepatocytes (Lap-tta-tetO-Wnt1) under the control of a tetracycline analog (doxycycline) as previously reported (9). Doxycycline water was given in all breeding cages and removed when the mice were 8 to 10 weeks old. After doxycycline withdrawal for 4 weeks, mice were used for experiments. To investigate the effects of Wnt/β-catenin signaling on hepatic steatosis, adult male Wnt1 overexpression transgenic mice (Wnt+) and wild-type (WT) mice were fed a HFD for 12 weeks. All experiments were conducted under a protocol approved by Stanford University School of Medicine Institutional Animal Care and in accordance with NIH guidelines.

### 2.2 Dietary treatments

To induce hepatic steatosis, mice at age of two months were fed free access to a HFD (BIO-SERV, F3282, Flemington, NJ) for 12 weeks before sacrifice for experiments (14, 15). Control mice were fed a standard control chow diet. Liver/body weight ratio was determined after sacrifice.

### 2.3 Hepatocellular function

To assess hepatocellular function, blood was obtained by cardiac puncture and serum was collected. Glucose, cholesterol, and triglyceride levels were determined using a standard clinical automatic analyzer.

### 2.4 Hematoxylin-eosin and oil red O staining

Liver tissues were fixed in 10% formalin, embedded in paraffin and sections were stained with hematoxylin-eosin (H&E). For oil red O (ORO) staining, cells were stained by incubation with 0.3% of filtered ORO solution in 60% isopropanol for 30 minutes at 37°C. The nuclei were counterstained with hematoxylin for 1 minute at room temperature. The cells were then photographed using phase-contrast microscopy. For quantification of intracellular lipids, cells were scraped by PBS and pelleted by spin down at 13000 rpm for 10 minutes after ORO staining. ORO stain was extracted by adding 0.5 ml of isopropanol for 30 minutes at room temperature. The absorbance of the ORO-containing solution was read at 520 nm using a microplate reader (SpectraMax i3x, Molecular Devices, San Jose, CA) (16). Experiments were performed in triplicate for data summary and statistical analysis.

### 2.5 Cell culture

The differentiated non-transformed mouse hepatocyte cell lines AML12 cells (American Type Culture Collection, Manassas, VA) and mutant AML12 hepatocytes carrying an amino terminus phosphorylation-resistant point mutation (β-catenin stabilized cells) (S33Y) were derived as previously reported (9) and cultured in DMEM/F12 medium (HyClone Thermal Fisher Scientific, Pittsburgh, PA) supplemented with 10% fetal calf serum at 37°C with 5% CO_2_. For induction of steatosis, cells were treated with 150 nM oleic acid (OA) for 24 hours followed by growth with normal medium overnight before being harvested for analysis. For mTOR inhibition, cells were treated with mTOR inhibitor rapamycin at a final concentration of 20 nM for one hour before being harvested for analysis. IWR-1-endo (IWR) was used as a Wnt/β-catenin inhibitor at a final concentration of 20 µM for pre-treatment of cells for 16 hours, and then continue to treat for additional 24 hours with or without 150 nM of OA. For activation of Wnt/β-catenin signaling, Wnt3a (50 ng/ml) and lithium chloride (LiCl, at 10 mM) were pre-incubated with AML12 cells for 16 hours respectively, then continue to treat the cells with or without 150 nM of OA for 24 hours.

### 2.6 Western blot analysis

Harvested cells after induction with or without OA for 24 hours or mouse liver samples were lysed in RIPA buffer containing protease inhibitor cocktails and sonicated for 10 seconds with the sonicator (Microson). 20μg of samples were added into each lane. Lysates were separated on SDS-polyacrylamide gels followed by immunoblotting with indicated primary antibodies at 4°C overnight according to standard protocol. The antibody Ab-α-tubulin served as a loading control. Reactive protein was visualized with the ECL kit (Amersham Pharmacia Biotech Inc, Piscataway, NJ) according to the manufacturer’s protocol. Signals were semi-quantified with Image J software (NIH.gov.USA).

### 2.7 Co-immunoprecipitation assay

For co-immunoprecipitation (co-IP) assays, cells were lysed in Nonidet P-40 extraction buffer and incubated with 1µg anti-mTOR antibody and protein A/G sepharose beads (Invitrogen, Camarillo, CA) at 4°C overnight. Bead-bound proteins were eluted by incubating in SDS-sample buffer at 95°C for 10 minutes and detected by immunoblotting using β-catenin antibody. Normal rabbit immunoglobulin G was used as a negative control.

### 2.8 Reagents

The following chemicals and reagents were used: DMEM/F12 medium (Hyclone, UT), fetal bovine serum (FBA), penicillin-streptomycin, trypsin/EDTA, protease inhibitor cocktails (Sigma, ST Louis, MO). ECL kit was from Amersham Bioscience. Other reagents used in this study were analytical grade and obtained from Sigma Chemical. The following antibodies were used in this study: β-catenin, sterol regulatory element binding protein 1(SREBP-1), medium chain acetyl coenzyme A dehydrogenase (MCAD) (Santa Cruz Biotechnology Inc, Santa Cruz, CA), Cyclin D1, phospho-SREBP-1c (Ser372), fatty acid synthase (FASN), acetyl coenzyme A carboxylase (ACC), phospho- acetyl coenzyme A carboxylase (p-ACC), acetyl coenzyme A synthetase (AceCS1), peroxisome proliferator-activated receptor γ (PPAR-γ), Akt, phospho-Akt (Ser437), phospho-Akt (Thr308), S6K, phospho-S6K (p-S6K), mTOR and α-tubulin (Cell Signalling Technology Inc, Danvers, MA), peroxisome proliferator-activated receptor α (PPAR-α) (Abcam, Cambridge, MA).

### 2.9 Statistics

Each group consisted of at least five animals unless otherwise indicated. Data are expressed as mean ± standard deviation and evaluated by Student’s t test (SPSS Statistics, version 19.0, Chicago). The data graphs were made with GraphPad Prism 5.0 software(Graph-Pad Software, CA). Significance was defined as p<0.05; “n.s.” indicates not significant.

## 3 Results

### 3.1 Hepatocyte-specific Wnt1 overexpression induces hepatic steatosis and obesity in HFD-fed mice

**Figure 1:**
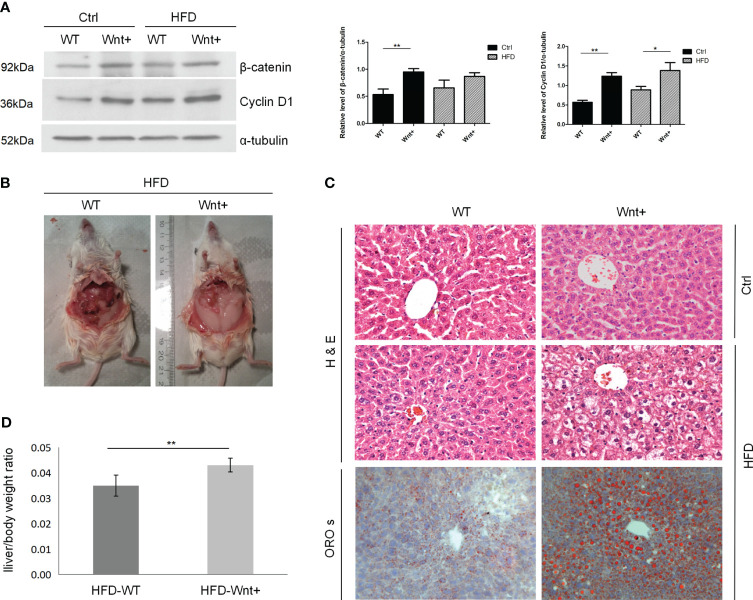
Wnt+ mice are more susceptible to liver steatosis under high fat diet. Wild-type and Wnt1 overexpressing (Wnt+) mice were fed with HFD or normal control diet for 12 weeks. **(A)** Immunoblot shows a significant increase in β-catenin and Cyclin D1 protein in Wnt+ livers. α-tubulin served as a loading control. Signals were semi-quantified with Image J software. **(B)** By abdominal inspection, Wnt+ mice demonstrated severe abdominal obesity. **(C)** Representative liver histology of untreated or HFD-treated livers. Severe hepatic steatosis is detected in Wnt+ mice after HFD. H&E staining reveals increased liver steatosis in β-catenin-stabilized livers with lipid accumulation and hepatocytes ballooning. Lipid droplets are detected by ORO staining. **(D)** The liver/body weight ratio was measured after HFD in WT and Wnt+ mice. A significantly increased liver/body weight ratio was observed in Wnt+ mice after HFD compared to WT controls. Data are expressed as means ± SD of three separate experiments. Ctrl control group, HFD high fat diet, WT Wild-type, Wnt+ Wnt1 overexpressing, H&E hematoxylin-eosin, ORO s oil red O staining. *p value<0.05, **p value<0.01.

Western blot (WB) analysis of proteins from liver tissue confirmed increased β-catenin and Cyclin D1 expression in Wnt+ mice compared to WT controls as a result of activated Wnt signaling (**Figure 1A**). By abdominal inspection, there was a grossly visible difference in adipose tissue deposits in the Wnt+ mice in response to HFD when compared to WT controls (**Figure 1B**). To assess the degree of steatosis induced by HFD, we performed H&E and ORO staining. In response to HFD, Wnt+ mice demonstrated increased steatosis with large vacuoles and lipid droplets consistent with macrovascular fatty changes (**Figure 1C**). In line with these findings, a significantly increased liver/body weight ratio in Wnt+ mice after HFD compared to WT controls (p=0.0037) (**Figure 1D**). Together these findings demonstrate that Wnt signaling activation in hepatocytes leads to hepatic steatosis under HFD.

### 3.2 Wnt signaling activation in hepatocytes increases fatty acid synthesis in HFD-fed mice liver

**Figure 2:**
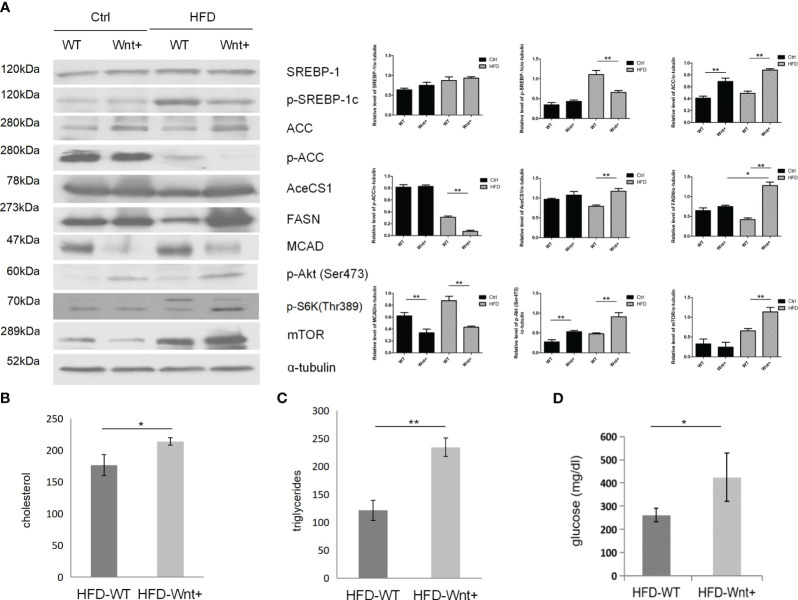
Increased fatty acid synthesis in Wnt+ mice under high fat diet. **(A)** Immunoblot shows a significant increase in the expression of fatty acid synthesis-related genes (SREBP-1 and its downstream targets ACC, AceCS1, and FASN) and a decrease in fatty acid oxidation gene (MCAD) in Wnt+ livers under HFD. mTOR and its downstream gene Phospho-S6K were significantly increased in Wnt+ mice in response to HFD. α-tubulin served as a loading control. Increased serum cholesterol **(B)**, triglycerides **(C)**, and glucose **(D)** levels were found in HFD-treated Wnt+ mice when compared to wildtype. WT Wild-type, HFD high fat diet. *p value<0.05, **p value<0.01.

Since Wnt-activated livers demonstrated severe steatosis, we questioned whether Wnt signaling might also regulate fatty acid synthesis in the liver. WB analysis was used to compare the expression of various genes involved in adipogenesis in Wnt+ and WT mice. Our results demonstrated that Wnt+ mice showed increased expression of SREBP-1 and its downstream targets ACC, AceCS1, and FASN after 12 weeks of HFD (**Figure 2A**, lane 4 vs. 3). Interestingly, Wnt+ mice demonstrated significantly impaired medium chain acyl-CoA dehydrogenase (MCAD) expression in both Ctrl and HFD groups (**Figure 2A**, lane 2 vs. 1 and lane 4 vs. 3) suggesting a failed beta-oxidation of medium-chain triglycerides. Taken together, these findings indicate that mice with activated Wnt signaling demonstrate increased lipogenesis and disrupted β-oxidation.

### 3.3 The Akt/mTOR pathway is up-regulated in Wnt+ HFD-fed mice

Wnt+ mice demonstrated increased expression of p-Akt at these two residues (**Figure 2A**, lane 2 vs. 1 and lane 4 vs. 3). mTOR, a target of Akt, was significantly increased in Wnt+ mice in response to HFD (**Figure 2A**, lane 4 vs. 3). Phospho-S6K, a downstream gene of mTOR, was also increased in Wnt+ mice in response to HFD (**Figure 2A**, lane 4 vs. 3). All these results indicate mTOR is an important modulator for steatosis in Wnt+ mice. To determine the effect of nutrient stress on Wnt-activated livers, we measured serum cholesterol and triglycerides in Wnt+ and WT mice. As a sign of increased fatty acid synthesis, cholesterol and triglyceride levels were significantly higher in the Wnt+ mice in response to HFD (p=0.015 and p=0.001, respectively.) (**Figures 2B, C**). Wnt+ mice also exhibited markedly elevated serum glucose levels in response to HFD (p=0.004) (**Figure 2D**).

### 3.4 β-catenin stabilization *in vitro* leads to increased lipid accumulation in hepatocytes with OA via mTOR signaling

**Figure 3:**
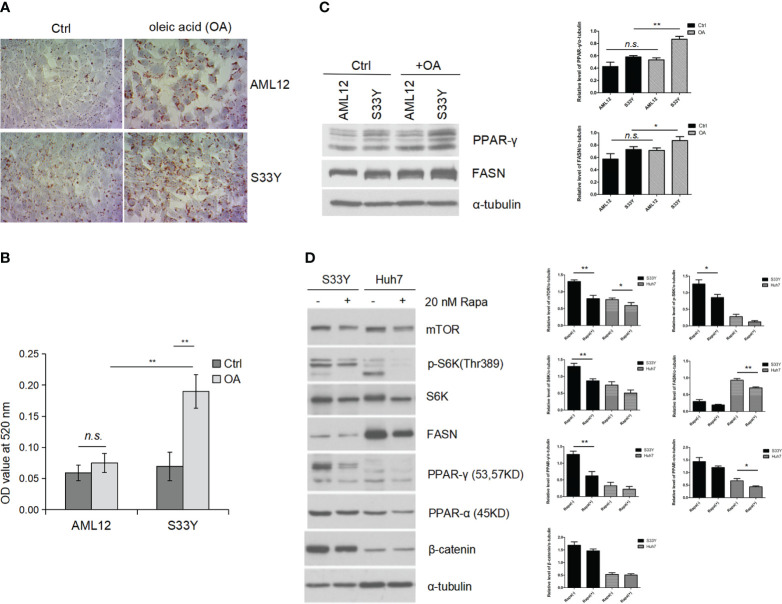
Activated Wnt/β-catenin signaling promotes steatosis *in vitro* under oleic acid. AML12 hepatocytes and β-catenin stabilized AML12 (S33Y) cells were treated with 150 nM OA for 24 hours for steatosis induction. **(A)** Increased lipid accumulation was detected in S33Y cells in response to OA treatment compared to AML12 cells as detected by ORO staining. **(B)** For quantification of intracellular lipids, ORO absorbance was measured by spectrophotometer. A significant increase of lipid accumulation in the OA-treated S33Y cells was observed compared to AML12 cells. **(C)** Increased fatty acid synthesis-related genes (PPRA γ, FASN) were up-regulated in S33Y hepatocytes after OA incubation for 24 hours. α-tubulin served as a loading control. **(D)** Cells were treated with the mTOR inhibitor rapamycin at 20 nM for one hour. Inhibition of mTOR led to decreased expression of p-S6K, PPAR-γ, and FASN as detected by immunoblot analysis. OA oleic acid. *p value<0.05, **p value<0.01, n.s., not significant.

S33Y cells demonstrated increased lipid accumulation with 150nM OA treatment as measured by ORO staining (**Figure 3A**) consistent with the steatotic phenotype seen in the *in vivo* model. The ORO dye was extracted from cells and quantified by absorbance reading which revealed a significant increase of lipid accumulation in the OA-treated S33Y cells compared to AML12 cells (0.19 ± 0.03 vs. 0.08 ± 0.02, p=0.0049) (**Figure 3B**). WB analysis demonstrates significantly increased expression of the fatty acid synthesis genes PPAR-γ and FASN in S33Y cells after OA treatment (**Figure 3C**, lane 4 vs. 2). However, this change was less pronounced in wildtype AML12 hepatocytes. (**Figure 3C**, lane 3 vs. 1).

To test whether increased fatty acid synthesis in S33Y cells is mediated through mTOR signaling, the mTOR inhibitor rapamycin (20nM) was added to interrogate its function as an upstream regulator. Inhibition of mTOR resulted in decreased expression of p-S6K, PPAR-γ, and FASN (**Figure 3D**, lane 3 vs. 4). Moreover, this similar trend is also shown in human huh7(hepatic cancer) cells (**Figure 3D**, lane 5 vs. 6).

### 3.5 β-catenin acts as an upstream regulator of mTOR in fatty acid synthesis.

**Figure 4:**
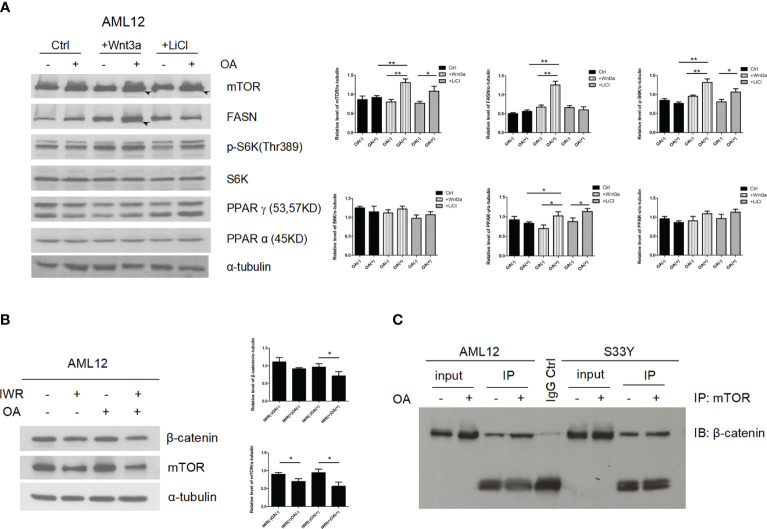
β-catenin directly interacts with mTOR signaling to induce lipogenesis. **(A)** Increased expression of lipid synthesis-related genes (mTOR, p-S6K, FASN, and PPAR-γ) were detected by immunoblotting in response to OA when Wnt/β-catenin signaling was activated via Wnt3a or LiCl application (lane 4 vs. 3 and 6 vs. 5). **(B)** After adding IWR (IWR-1-endo, an inhibitor of Wnt-signaling) to AML12 cells, the expression of β-catenin and mTOR was down-regulated (lane 2 vs. 1 and 4 vs. 3). In addition, after Wnt signaling was inhibited, even under the effect of OA, the expression level of mTOR did not increase (lane 4 vs. 2). **(C)** β-catenin has a physical interaction with mTOR, which was verified by co-immunoprecipitation in hepatocytes using anti-mTOR antibody followed by β-catenin immunoblotting. LiCl lithium chloride. Data were shown as mean ± SD of three separate experiments. *P < 0.05, **P < 0.01, based on a Student’s t-test.

In order to test whether the effect of β-catenin stabilization on mTOR and fatty acid synthesis can be reproduced through Wnt signaling activation to mimic the *in vivo* findings, the Wnt signaling agonists, Wnt3a and LiCl were added to AML12 cells. In the control (Ctrl) group, even administration of OA did not affect the changes in the expression of lipid synthesis-related gene (mTOR, p-S6K, FASN, and PPAR-γ) (**Figure 4A**, lane 1 vs. 2). In contrast, in the Wnt3a and LiCl groups, the expression level of lipid synthesis-related genes under the effect of OA was higher than that of levels without OA (**Figure 4A**, lane 4 vs. 3 and 6 vs. 5). In addition, under the same condition of OA, the expression level of lipid synthesis-related genes in Wnt3a and LiCl groups was higher than that in Ctrl group (**Figure 4A**, lane 4 vs.2 and 6 vs.2).

After adding IWR (IWR-1-endo, an inhibitor of Wnt-signaling) to AML12 cells, the expression of β-catenin and mTOR was down-regulated (**Figure 4B**, lane 2 vs. 1 and 4 vs. 3). In addition, after Wnt signaling was inhibited, even under the effect of OA, the expression level of mTOR and FASN did not increase (**Figure 4B**, lane 4 vs. 2). To further investigate if the activation of mTOR is a result of binding to β-catenin, co-IP assays were performed. The results reveal that β-catenin has a physical interaction with mTOR (**Figure 4C**). These findings suggest that fatty acids can induce and promote formation of β-catenin/mTOR complex.

**Figure 5:**
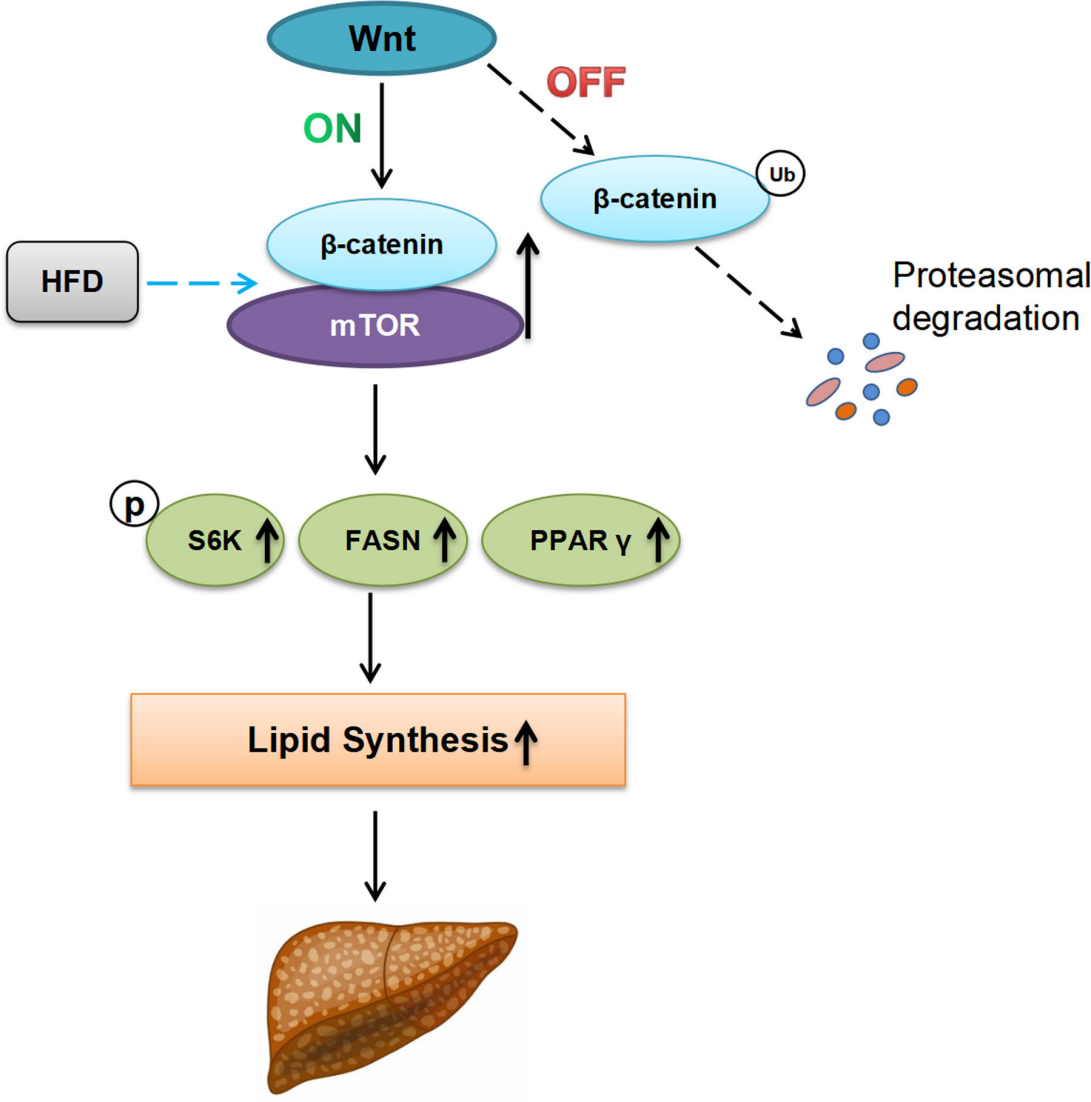
Proposed working model describing the possible role of Wnt/β-catenin signaling for hepatosteatosis. In the absence of Wnt-signal (Off-state), ubiquitination and proteasome degradation of β-catenin will occur. Under this condition, the effect of HFD on hepatic fat synthesis is minimal. In the presence of Wnt ligand (On-state), HFD can induce and promote the formation of β-catenin/mTOR complex in the liver, leading to the upregulation of lipid synthesis-related genes (p-S6K, FASN, and PPAR-γ), increasing the lipid synthesis, and the developing of hepatic steatosis finally. Ub ubiquitination, HFD high fat diet, P phosphorylation.

Taken together these data support a model in which Wnt/β-catenin regulate lipid metabolism in the steatotic liver and function as a molecular regulator for fatty acid synthesis via Akt/mTOR signaling (**Figure 5**).

## 4 Discussion

NAFLD, characterized by fat accumulation in the liver, is a wide spectrum of liver diseases ranging from simple fatty liver to non-alcoholic steatohepatitis (NASH), which may progress further to end-stage liver diseases like cirrhosis and hepatocellular carcinoma (17). Behari J, et al. reported that HFD-fed hepatocyte-specific β-catenin transgenic mice rapidly developed diet-induced obesity. Canonical Wnt signaling in hepatocytes is essential for the development of diet-induced fatty liver and obesity (4). On the contrary, reducing β-catenin expression decreases the expression of enzymes involved in hepatic fatty acid esterification, and ameliorates hepatic steatosis (5). In recent years, activation of the Wnt/β-catenin pathway was found to increase lipogenesis in HepG2 cells via regulation of endoplasmic reticulum stress (6). In the present study, we found hepatocyte-specific Wnt1 overexpression induces hepatic steatosis and obesity in HFD-fed mice. However, in a previous study, β-Catenin was considered to act as an anti-adipogenic factor to inhibit the expression of PPAR-γ (18). Therefore, the effects of the Wnt/b-catenin signaling pathway on lipid metabolism are complicated.

Our results demonstrated a significant increase in the expression of fatty acid synthesis-related genes (SREBP-1 and its downstream targets ACC, AceCS1, and FASN) and a decrease in fatty acid oxidation gene (MCAD) in Wnt+ HFD-fed mice livers. SREBP-1 is a master regulator of lipid metabolism and activates a wide range of lipid genes including ACC, acetyl AceCS1, and FASN by binding promoter sites termed sterol regulatory elements (19). MCAD is one of the significant enzymes involved in the β-oxidation of mitochondrial fatty acids. The previous study found that the MCAD levels in the liver were significantly reduced in NASH patients compared to patients without NASH. Moreover, upregulation of MCAD expression also reduced lipid deposition and improved NASH *in vivo* and *in vitro* (20). Therefore, based on the above results, it is concluded that hepatic fat accumulation in Wnt+ HFD-fed mice may be due to increased lipid synthesis and decreased oxidative catabolism caused by Wnt/β-catenin signaling activation. In addition, we also found that Wnt+ mice demonstrated increased expression of p-Akt (both Ser473 and Thr308). mTOR, a target of Akt, was also significantly increased in Wnt+ mice in response to HFD. These results indicate that the Akt/mTOR pathway is up-regulated in Wnt+ mice with HFD.

To further explore the role and specific mechanisms of Wnt/β-catenin signaling in hepatic steatosis, we selected S33Y and AML12 cells for *in vitro* assays. The results of oil-red O staining demonstrated that there were more lipid droplets in the S33Y cells treated with OA. Moreover, Our result showed that the expression of PPAR-γ and FASN was significantly increased in OA-treated S33Y cells compared to AML12. PPAR-γ is a key regulator of adipogenesis (21). It is well known that PPAR-γ is a key regulator of insulin sensitivity and adipocyte differentiation (22). Previous studies have also shown increased PPAR-γ expression in mice that are fed a high-fat diet to induce hepatic steatosis (23).FASN is an essential enzyme that catalyzes the *de novo* synthesis of long-chain fatty acids (24). Thus, we have again demonstrated *in vitro* that Wnt/β-catenin signaling activation promotes lipogenesis in hepatocytes.

Deregulated mTOR signaling is implicated in the progression of cancer and diabetes, as well as the aging process (25). Moreover, activation of mTOR is associated with increased lipid synthesis and lipid droplets (12). This function is similar to the activation of Wnt/β-catenin signaling found in our study, so we speculate that mTOR signaling may interact with the Wnt/β-catenin pathway in some way. In the present study, our results showed that the expression of both mTOR and its downstream lipid metabolism-related target genes was elevated when Wnt/β-catenin signaling agonists were used. In contrast, mTOR expression was downregulated with the use of Wnt/β-catenin signaling inhibitors. In addition, β-catenin has a physical interaction with mTOR, which was verified by co-immunoprecipitation in hepatocytes. With this study, we extend previous findings and present that β-catenin specifically is important for mTOR signal activation in the liver.

We provide the first evidence of β-catenin directly binding to mTOR to increase fatty acid synthesis in the setting of HFD. However, mTOR complex 1 (mTORC1) signaling has been reported to suppress canonical Wnt/β-catenin signaling to influence stem cell maintenance (26). This indicates that we have not fully discovered its specific mechanism. In addition, we only found that β-catenin and mTOR are linked in terms of physical binding, but the specific mechanism is not fully understood, and more experiments are still needed to confirm it in the future.

## 5 Conclusion

In summary, we demonstrate that hepatocyte-specific Wnt1 overexpressing mice fed by HFD develop hepatic steatosis and obesity accompanied by increased lipogenesis and disrupted β-oxidation as well as augmented Akt/mTOR signaling. β-catenin stabilization *in vitro* leads to increased lipid accumulation in hepatocytes via mTOR crosstalk. Taken together, β-catenin stabilization through Wnt signaling serves a central role in lipid metabolism in the steatotic liver through up-regulation of fatty acid synthesis via Akt/mTOR signaling. These findings suggest hepatic Wnt signaling may represent a therapeutic strategy in metabolic liver disease.

## Data availability statement

The original contributions presented in the study are included in the article/supplementary material. Further inquiries can be directed to the corresponding author.

## Ethics statement

Ethical approval was not required for the studies on humans in accordance with the local legislation and institutional requirements because only commercially available established cell lines were used. The animal study was approved by Stanford University School of Medicine Institutional Animal Care. The study was conducted in accordance with the local legislation and institutional requirements.

## Author contributions

KW: Writing – original draft. RZ: Writing – review & editing. NL: Writing – original draft. G-ZT: Writing – review & editing. BWL: Writing – review & editing. BL: Writing – review & editing. YK: Writing – review & editing. KS: Writing – review & editing.
